# Intra‐ and interobserver agreement of proposed objective transvaginal ultrasound image‐quality scoring system for use in artificial intelligence algorithm development

**DOI:** 10.1002/uog.29178

**Published:** 2025-01-24

**Authors:** A. Deslandes, J. C. Avery, H.‐T. Chen, M. Leonardi, S. Knox, G. Lo, R. O'Hara, G. Condous, M. L. Hull, Collaborators, C. Panuccio, S. Maple, D. Petashvili

**Affiliations:** ^1^ Robinson Research Institute University of Adelaide Adelaide Australia; ^2^ School of Computer and Mathematical Sciences University of Adelaide Adelaide Australia; ^3^ Department of Obstetrics and Gynecology McMaster University Hamilton ON Canada; ^4^ Benson Radiology Adelaide Australia; ^5^ Sir Charles Gairdner Hospital Nedlands Western Australia Australia; ^6^ Curtin University Bentley Western Australia Australia; ^7^ Endometriosis Ultrasound & Advanced Endosurgery Unit, Sydney Medical School Nepean University of Sydney, Nepean Hospital Sydney Australia

**Keywords:** artificial intelligence, gynecology, image quality, interobserver agreement, intraobserver agreement, scoring system, transvaginal ultrasound

## Abstract

**Objectives:**

The development of valuable artificial intelligence (AI) tools to assist with ultrasound diagnosis depends on algorithms developed using high‐quality data. This study aimed to test the intra‐ and interobserver agreement of a proposed image‐quality scoring system to quantify the quality of gynecological transvaginal ultrasound (TVS) images, which could be used in clinical practice and AI tool development.

**Methods:**

A proposed scoring system to quantify TVS image quality was created following a review of the literature. This system involved a score of 1–4 (2 = poor, 3 = suboptimal and 4 = optimal image quality) assigned by a rater for individual ultrasound images. If the image was deemed inaccurate, it was assigned a score of 1, corresponding to ‘reject’. Six professionals, including two radiologists, two sonographers and two sonologists, reviewed 150 images (50 images of the uterus and 100 images of the ovaries) obtained from 50 women, assigning each image a score of 1–4. The review of all images was repeated a second time by each rater after a period of at least 1 week. Mean scores were calculated for each rater. Overall interobserver agreement was assessed using intraclass correlation coefficient (ICC), and interobserver agreement between paired professionals and intraobserver agreement for all professionals were assessed using weighted Cohen's kappa and ICC.

**Results:**

Poor levels of interobserver agreement were obtained between the six raters for all 150 images (ICC, 0.480 (95% CI, 0.363–0.586)), as well as for assessment of the uterine images only (ICC, 0.359 (95% CI, 0.204–0.523)). Moderate agreement was achieved for the ovarian images (ICC, 0.531 (95% CI, 0.417–0.636)). Agreement between the paired sonographers and sonologists was poor for all images (ICC, 0.336 (95% CI, −0.078 to 0.619) and 0.425 (95% CI, 0.014–0.665), respectively), as well as when images were grouped into uterine images (ICC, 0.253 (95% CI, −0.097 to 0.577) and 0.299 (95% CI, −0.094 to 0.606), respectively) and ovarian images (ICC, 0.400 (95% CI, −0.043 to 0.669) and 0.469 (95% CI, 0.088–0.689), respectively). Agreement between the paired radiologists was moderate for all images (ICC, 0.600 (95% CI, 0.487–0.693)) and for their assessment of uterine images (ICC, 0.538 (95% CI, 0.311–0.707)) and ovarian images (ICC, 0.621 (95% CI, 0.483–0.728)). Weak‐to‐moderate intraobserver agreement was seen for each of the raters with weighted Cohen's kappa ranging from 0.533 to 0.718 for all images and from 0.467 to 0.751 for ovarian images. Similarly, for all raters, the ICC indicated moderate‐to‐good intraobserver agreement for all images overall (ICC ranged from 0.636 to 0.825) and for ovarian images (ICC ranged from 0.596 to 0.862). Slightly better intraobserver agreement was seen for uterine images, with weighted Cohen's kappa ranging from 0.568 to 0.808 indicating weak‐to‐strong agreement, and ICC ranging from 0.546 to 0.893 indicating moderate‐to‐good agreement. All measures were statistically significant (*P* < 0.001).

**Conclusion:**

The proposed image quality scoring system was shown to have poor‐to‐moderate interobserver agreement and mostly weak‐to‐moderate levels of intraobserver agreement. More refinement of the scoring system may be needed to improve agreement, although it remains unclear whether quantification of image quality can be achieved, given the highly subjective nature of ultrasound interpretation. Although some AI systems can tolerate labeling noise, most will favor clean (high‐quality) data. As such, innovative data‐labeling strategies are needed. © 2025 The Author(s). *Ultrasound in Obstetrics & Gynecology* published by John Wiley & Sons Ltd on behalf of International Society of Ultrasound in Obstetrics and Gynecology.

## INTRODUCTION

Accurate interpretation of ultrasound images is reliant upon the acquisition of high‐quality images. Artificial intelligence (AI) algorithms may interpret ultrasound images, yielding a proposed diagnosis, but they are similarly dependent upon high‐quality image acquisition, with poor image quality (IQ) reducing the accuracy of diagnosis^1^, ^2^, ^3^. For instance, Blaivas *et al*.^2^ reported that the performance of deep learning systems reduced significantly when applied to images from low‐cost ultrasound machines with lower IQ. Therefore, it is prudent to consider AI tools to assess IQ, concurrent with the development of AI algorithms for diagnosis, ensuring AI systems receive data input of satisfactory quality to allow for accurate diagnosis.

Many scoring systems within the published literature propose ways of critically evaluating ultrasound IQ and allocating examinations a score for natural (human‐led) imaging^4^, ^5^, ^6^, ^7^, ^8^, ^9^, ^10^, ^11^, ^12^, ^13^, ^14^, ^15^, ^16^, ^17^, ^18^. However, many of these systems rate factors that are less relevant when developing AI tools, such as operator skill^4^, ^8^, ^11^ and accuracy of annotations^7^, ^9^, ^13^. Two systems specific to gynecological ultrasound have been published previously that focus on antral follicle counting^6^ and emergency gynecology presentations^11^, but there is currently no scoring system within the published literature for the assessment of transvaginal ultrasound (TVS) IQ.

AI algorithms have been developed to automate IQ assessment in medical imaging^19^, ^20^, ^21^, ^22^. Although such tools can be utilized solely for thresholding, by identifying and then rejecting poor‐quality images^23^, a broader IQ assessment system has the potential to provide wider clinical assistance. Chen *et al*.^24^ developed an AI system to classify intravascular ultrasound images as low, medium or high quality, which achieved a classification accuracy of 96.34%. This system was trained using a library of images whose quality was labeled subjectively by doctors, highlighting the need for suitable IQ labeling strategies in AI algorithm development. Additionally, such systems should be ideally trained on a full spectrum of data (ranging from high to low quality) to learn how to identify good *vs* poor IQ. This study aimed to propose a scoring system able to quantify the IQ of gynecological TVS images suitable for AI algorithm development and to assess the intra‐ and interobserver agreement of this tool among multiple ultrasound professionals.

## METHODS

### Development of scoring system

A search of the online databases PubMed, Scopus and Google Scholar was performed from inception to 30 October 2022 to ascertain whether a suitable scoring system for TVS IQ assessment exists. No such tool was found, and subsequently, a review of the literature was conducted to identify any other published IQ assessment tools for ultrasound imaging. The same online databases were searched again from inception to 30 October 2022 using the terms ‘ultrasound’, ‘scoring’ OR ‘grading’, ‘assessment’ and ‘quality’. Returned items were screened by title and abstract for relevance. Those considered likely to be relevant were then read in full. This ultimately revealed 15 studies detailing scoring systems related to ultrasound imaging (Table 1)^4^, ^5^, ^6^, ^7^, ^8^, ^9^, ^10^, ^11^, ^12^, ^13^, ^14^, ^15^, ^16^, ^17^, ^18^. Based on these identified systems published previously, a new draft scoring system based on anatomy demonstration and technical optimization of images was created to quantify IQ assessment of gynecological TVS images. This system was then reviewed by four experts in gynecological ultrasound (G.C., M.L., S.K. and C.P.) with discussion around the suitability of each score until agreement was reached on score descriptions. The draft scoring system was then reviewed by an expert in the development of AI tools for medical imaging (H.‐T.C.) to ensure its suitability for use in the design of AI tools. A final review from all experts was then conducted to create the new scoring system. Details of the various iterations of the scoring system throughout this process are provided in Appendix S1.

**Table 1:** Summary of 15 published studies on ultrasound image‐quality (IQ) scoring systems identified through literature search

		Scoring system	
Study	Title	Application	Summary
Bahner (2011)^8^	Brightness mode quality ultrasound imaging examination technique (B‐QUIET): quantifying quality in ultrasound imaging	General ultrasound	Allocates score of 1–4 for various aspects of scan anatomy, optimization and documentation.
Boelig (2017)^15^	Assessment of transvaginal ultrasound cervical length image quality	Cervical length	Assesses nine features specific to cervical length assessment. A score of 7/9 is required for the image to be deemed passable.
Dessie (2023)^17^	Development and validation of a point‐of‐care‐ultrasound image quality assessment tool: the POCUS IQ scale	POCUS	Allocates a score of 0–2 to various aspects of examination to a total of 14 points. Relates to labeling, optimization, probe selection, anatomy and measurements.
Iared (2018)^7^	Reproducibility of a quantitative system for assessing the quality of diagnostic ultrasound	Diagnostic ultrasound and report quality	Quantitative System for Assessing the Quality of Ultrasound (SQUALUS) Examinations. Assesses the technical quality of images and allocates a score of 0–10. Assesses the accuracy of reports and allocates a score of 0–10. The arithmetic mean of scores is then calculated to give a total score of 0–10.
Jayaprakasan (2007)^6^	The interobserver reliability of off‐line antral follicle counts made from stored three‐dimensional ultrasound data: a comparative study of different measurement techniques	Antral follicle counting	Allocates a grade of 1–3 (good, medium or poor) based on the amount of ovarian contour seen in the image.
Lam (2015)^9^	Multi‐institution validation of an emergency ultrasound image rating scale—a pilot study	Emergency medicine	Allocates a score of 1–5 for landmarks, 1–3 for IQ (optimization) and 1–2 for accuracy of annotations to a total of 10. A score of 3–10 is considered acceptable.
Liu (2018)^14^	Emergency ultrasound standard reporting guide; American College of Emergency Physicians	Emergency medicine	Five‐point scale ranging from ‘no recognizable features’ to ‘excellent’. A score of 1–5 is allocated to represent IQ.
Millington (2016)^5^	The rapid assessment of competency in echocardiography scale: validation of a tool for point‐of‐care ultrasound	Echocardiography	Allocates a score of 0–5 for each specific view where 0 is not obtained, 1 is too poor to draw meaningful information, 3 is a suboptimal image but basic interpretation possible and 5 is a good image with easy interpretation.
Molloholli (2019)^10^	Image‐scoring system for umbilical and uterine artery pulsed‐wave Doppler ultrasound measurement	Obstetric Doppler	Doppler traces were graded as acceptable *vs* unacceptable based on six technical features, with one point allocated for each. A score of four or more was deemed acceptable.
Qiu (2021)^13^	Value of ultrasound images and reports scoring system in quality control	General ultrasound	A score of 0–5 was allocated relating to IQ, optimization (B‐mode and Doppler) and accuracy of annotations. A score of 0–5 was allocated to scan reports, assessing for inaccuracies. A score of 3 or less was considered a failure.
Salomon (2006)^12^	Feasibility and reproducibility of an image‐scoring method for quality control of fetal biometry in the second trimester	Fetal biometry	One point for visualizing multiple anatomical structures and technical aspects of scans. Assesses femur length, abdominal circumference and biparietal diameter.
Salomon (2009)^11^	A score‐based method to improve the quality of emergency gynaecological ultrasound examination	Gynecological scans in emergency medicine	Focuses on acquiring standard key images with one point assigned for each feature seen in scans to a total of 23 points. Morrison's pouch is included as a view, as this can be relevant to acute gynecological cases.
Tolsgaard (2013)^16^	International multispecialty consensus on how to evaluate ultrasound competence: a Delphi consensus survey	Diagnostic ultrasound	Developed by Delphi consensus. Assesses operator skills in terms of both technical ability and clinical knowledge across any ultrasound being performed (e.g. obstetric, breast, abdominal and gynecological). Comprises seven elements related to performing ultrasound examination.
Tolsgaard (2014)^18^	Reliable and valid assessment of ultrasound operator competence in obstetrics and gynecology	Obstetrics and gynecology	Assesses five criteria using a 5‐point Likert scale. Assesses both image acquisition and interpretation.
Ziesmann (2015)^4^	Validation of the quality of ultrasound imaging and competence (QUICk) score as an objective assessment tool for the FAST examination	eFAST scanning	Developed by Delphi consensus to be objective. Assesses multiple competencies related to eFAST scanning on the task‐specific checklist to assign competence.

Only the first author is given for each study.

eFAST, extended focused assessment with sonography for trauma; POCUS, point‐of‐care ultrasound.

### Proposed scoring system for assessment of TVS IQ

**Figure 1:**
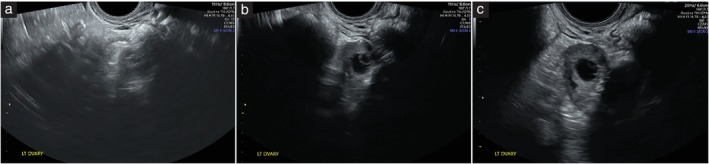
Example of ovarian ultrasound images with poor image quality (IQ) (score 2) (a), suboptimal IQ (score 3) (b) and optimal IQ (score 4) (c). A score of 1 was allocated to images deemed incorrect; these images were then rejected. (a) It is difficult to use this image for diagnosis as the ovary cannot be identified confidently, with the entire ovary not seen in the image, and optimization is poor. As the overall IQ is poor, a score of 2 is assigned. (b) The ovary can be seen. With the entire ovary visualized, optimization is satisfactory, but it could be improved, and diagnosis should be possible from the image. As the overall IQ is satisfactory, a score of 3 is assigned. (c) The entire ovary is documented clearly, optimization is good, and this image can be interpreted easily for diagnosis. As the overall IQ is good, a score of 4 is assigned. Lt, left.

The proposed scoring system is given in Table 2. If an image was deemed incorrect/inaccurate (e.g. blank image or transabdominal rather than transvaginal image required), the image was rejected (score of 1). All other images were assessed against the five factors listed in Table 2 relating to the following: correct depiction of anatomy; view of the anatomical structure in the field of view; image optimization (depth, focus and gain); ability to interpret image for diagnosis of pathology; and overall clarity of the image. A score of 1–4 that, in the opinion of the rater, best represented the image, was allocated for each image, where 1 = reject (image incorrect), 2 = poor IQ, 3 = suboptimal IQ and 4 = optimal IQ. A selection of images representing scores 2–4 are shown in Figure 1.

**Table 2:** Proposed transvaginal ultrasound image‐quality (IQ) scoring system

Reject: image inaccurate (score 1)	Poor IQ (score 2)	Suboptimal IQ (score 3)	Optimal IQ (score 4)
N/A	Correct anatomy not confidently recognizable	Correct anatomy recognizable	Correct anatomy easily recognizable
N/A	Part of the anatomical structure not seen	Most of the anatomical structure seen clearly	Entire anatomical structure seen clearly
N/A	Unacceptable image optimization (depth, focus and gain)	Acceptable image optimization (depth, focus and gain)	Good image optimization (depth, focus and gain)
N/A	Unable to interpret the image for diagnosis	Possible to interpret the image for diagnosis	Easy to interpret the image for diagnosis
N/A	Overall image clarity is poor	Overall image clarity is satisfactory	Overall image clarity is good

Images are assessed by the five factors listed and assigned a score of 1–4 that best represents the image; a score of 4 represents optimal IQ while a score of 2 represents poor IQ.

N/A, not applicable.

### Intra‐ and interobserver reliability

To assess the intra‐ and interobserver reliability, IQ scoring was performed independently by six imaging professionals, paired by specialization: two gynecologists with a subspecialization in ultrasound (gynecological sonologists) (G.C. and M.L.), two sonographers (C.P. and S.M.) and two radiologists with subspecialization in gynecological imaging (S.K. and G.L.). All professionals have a high level of expertise in the performance and/or offline interpretation of TVS images, with a minimum experience of 6 years.

**Figure 2:**
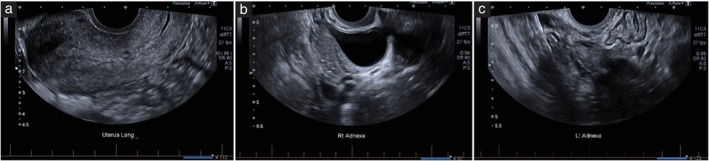
Example of one case (three ultrasound images) reviewed by raters. For each case, ultrasound images in the midsagittal plane of the uterus (a), right ovary (b) and left ovary (c) were reviewed. All images reviewed were single‐screen images, free of measurement calipers and color Doppler boxes. Anatomical annotations remained on images; however, information that could identify the patient or clinic from which the image was acquired was removed before starting the study. Lt, left; Rt, right.

There were 50 cases contributing three still images each (no cineclips), totaling 150 images for the image review. The images reviewed and scored were midsagittal images of the uterus (*n* = 50), the right ovary (*n* = 50) and the left ovary (*n* = 50) (Figure 2). The images were obtained from a large, retrospectively collected data library obtained as part of a wider study, the IMAGENDO® study (trial registration number: ACTRN12623000646640). Images were from a range of ultrasound clinics in Australia and were acquired on ultrasound machines from various equipment manufacturers. All images reviewed were two‐dimensional B‐mode single‐screen images, free of measurement calipers and color Doppler boxes. Images did not contain any data related to patient identifiers, such as the clinic in which the image was obtained or the date of the examination. Details of the ultrasound machine used to obtain the images were removed from the images as much as was practicable. The reviewed images were prepared by an independent investigator (A.D.). The reviewers were blinded to any other images from the scan, clinical history, sonographer's report (i.e. technical limitations of the real‐time scan) or formal examination report. Reviewed images were uploaded to an online survey platform (Survey Monkey, 2023; Momentive.ai, San Mateo, CA, USA) for data capture. Within Survey Monkey, each of the six raters reviewed every image and allocated a score for each one, according to the scoring system in Table 2, from a multiple‐choice selection. All images were viewed by each rater in unique locations and on personal devices. A free‐text box was included to capture any comments the raters had concerning any technical limitations they believed may have contributed to IQ. If a score of 1, 2 or 3 was assigned, raters were instructed to add free‐text comments detailing factors they believe may have contributed to reduced IQ. The image review process was repeated by each rater after an interval of at least 1 week, for a total of two scores per image for each rater. This study was conducted as part of the IMAGENDO® study for which ethics approval was obtained from the University of Adelaide Human Research Ethics Committee (protocol number: H‐2020‐051).

### Statistical analysis

Interobserver agreement was assessed using the intraclass correlation coefficient (ICC), which was calculated to measure levels of agreement between all six raters, where 0 = no agreement and 1 = perfect agreement (< 0.5 = poor agreement, 0.5–0.75 = moderate agreement, 0.75–0.9 = good agreement and 0.9 = excellent agreement)^25^. ICC estimates were based on a single rater value of k = 6, indicating absolute agreement, using a two‐way mixed‐effects model. The interobserver agreement of the paired professionals was also assessed using ICC and weighted Cohen's kappa coefficient, where kappa < 0.20 = no agreement, 0.21–0.39 = minimal agreement, 0.40–0.59 = weak agreement, 0.60–0.79 = moderate agreement, 0.80–0.90 = strong agreement and > 0.90 = almost perfect agreement^26^.

Median, mode and mean scores from each rater at each review session were calculated. Weighted Cohen's kappa and ICC values were calculated to assess the intraobserver agreement for each rater, over the two review sessions. A biostatistician was consulted to verify that this analysis method was satisfactory. SPSS for Mac version 29.0.2.0 (IBM Corp., Armonk, NY, USA) was used for ICC calculations. A *P*‐value < 0.05 was considered statistically significant. A sample size calculation was performed using an online sample size calculator (http://wnarifin.github.io), which confirmed that a minimum sample of 45 images was required for a minimum acceptable ICC of 0.5 and an expected ICC of 0.75 with a significance level of 0.05 to achieve a power of 80%^27^.

## RESULTS

### Interobserver agreement

Table 3 presents the interobserver agreement achieved among the six raters and that achieved when raters were paired by professional specialization. Poor levels of agreement were obtained between the six raters for all 150 images (ICC, 0.480 (95% CI, 0.363–0.586)), as well as when images were grouped into uterine images (ICC, 0.359 (95% CI, 0.204–0.523)). Moderate levels of agreement between all raters were achieved for images of the ovaries (ICC, 0.531 (95% CI, 0.417–0.636)). Agreement between the paired sonographers and sonologists was poor for all images (ICC, 0.336 (95% CI, −0.078 to 0.619) and 0.425 (95% CI, 0.014–0.665), respectively), as well as when images were grouped into uterine images (ICC, 0.253 (95% CI, −0.097 to 0.577) and 0.299 (95% CI, −0.094 to 0.606), respectively) and ovarian images (ICC, 0.400 (95% CI, −0.043 to 0.669) and 0.469 (95% CI, 0.088–0.689), respectively). Moderate levels of agreement were achieved between the paired radiologists for all images overall (ICC, 0.600 (95% CI, 0.487–0.693)) and for images of the uterus (ICC, 0.538 (95% CI, 0.311–0.707)) and ovaries (ICC, 0.621 (95% CI, 0.483–0.728)). All measures were statistically significant with a *P*‐value < 0.001.

**Table 3:** Interobserver agreement among all six raters and according to their professional group for assessment of image quality of 150 ultrasound images, overall and grouped by uterine and ovarian images

	All (*n* = 150)		Uterine (*n* = 50)		Ovarian (*n* = 100)	
Rater	ICC	Cohen's kappa	ICC	Cohen's kappa	ICC	Cohen's kappa
All (*n* = 6)	0.480 (0.363–0.586)	—	0.359 (0.204–0.523)	—	0.531 (0.417–0.636)	—
Radiologists (*n* = 2)	0.600 (0.487–0.693)	0.480 (0.374–0.587)	0.538 (0.311–0.707)	0.491 (0.269–0.714)	0.621 (0.483–0.728)	0.471 (0.350–0592)
Sonographers (*n* = 2)	0.336 (−0.078 to 0.619)	0.214 (0.143–0.284)	0.253 (−0.097 to 0.577)	0.109 (0.034–0.184)	0.400 (−0.043 to 0.669)	0.277 (0.179–0.376)
Sonologists (*n* = 2)	0.425 (0.014–0.665)	0.235 (0.155–0.314)	0.299 (−0.094 to 0.606)	0.094 (0.006–0.183)	0.469 (0.088–0.689)	0.287 (0.189–0.386)

Values in parentheses are 95% CI.

ICC, intraclass correlation coefficient.

### Intraobserver agreement

Intraobserver agreement is shown in Table 4. Weighted Cohen's kappa revealed weak‐to‐moderate agreement for each of the raters for all images (Cohen's kappa ranged from 0.533 to 0.718) and for images of the ovaries (Cohen's kappa ranged from 0.467 to 0.751). Slightly better agreement was seen for uterine images, with weighted Cohen's kappa ranging from 0.568 to 0.808, indicating weak‐to‐strong agreement. The ICC revealed similar results, with moderate‐to‐good intraobserver agreement for all raters for all images overall (ICC ranged from 0.636 to 0.825), as well as for images of the uterus (ICC ranged from 0.546 to 0.893) and ovaries (ICC ranged from 0.596 to 0.862). All measures were statistically significant with a *P*‐value < 0.001.

**Table 4:** Intraobserver agreement of six raters for assessment of image quality of 150 ultrasound images, overall and grouped by uterine and ovarian images

	All (*n* = 150)		Uterine (*n* = 50)		Ovarian (*n* = 100)	
Rater	ICC	Cohen's kappa	ICC	Cohen's kappa	ICC	Cohen's kappa
M.L.	0.819 (0.757–0.866)	0.718 (0.627–0.809)	0.579 (0.359–0.738)	0.591 (0.383–0.800)	0.862 (0.797–0.907)	0.751 (0.655–0.846)
G.C.	0.636 (0.530–0.722)	0.533 (0.409–0.657)	0.733 (0.573–0.839)	0.676 (0.483–0.868)	0.596 (0.454–0.708)	0.467 (0.311–0.623)
S.M.	0.703 (0.561–0.796)	0.613 (0.515–0.712)	0.773 (0.628–0.865)	0.674 (0.523–0.825)	0.662 (0.464–0.784)	0.579 (0.452–0.706)
C.P.	0.770 (0.693–0.829)	0.708 (0.607–0.809)	0.546 (0.321–0.713)	0.568 (0.338–0.799)	0.807 (0.725–0.866)	0.736 (0.626–0.846)
G.L.	0.729 (0.645–0.796)	0.589 (0.495–0.684)	0.729 (0.566–0.837)	0.589 (0.398–0.780)	0.717 (0.607–0.800)	0.575 (0.463–0.687)
S.K.	0.825 (0.765–0.870)	0.697 (0.619–0.775)	0.893 (0.819–0.938)	0.808 (0.696–0.919)	0.794 (0.706–0.857)	0.645 (0.544–0.746)

Values in parentheses are 95% CI.

*P* < 0.001 was achieved for all measures.

ICC, intraclass correlation coefficient.

Table S1 shows the median, mode and mean scores assigned by each rater at the first and second review sessions. Overall, the level of criticism exercised by the raters was consistent across both sessions. However, three raters (G.C., S.M. and C.P.) assigned slightly higher mean scores across both the uterine and ovarian images on their second review. The spread of mean scores between raters across the two sessions (first review: mean scores ranged from 2.6 to 3.5; second review: mean scores ranged from 2.8 to 3.5) suggests that certain raters were more critical of IQ while other raters were more lenient.

## DISCUSSION

This study revealed that the interobserver agreement for assigning quantitative scores to TVS images using our scoring system ranged from poor to moderate. Meanwhile, intraobserver reliability was slightly better, demonstrating moderate‐to‐good ICC and weak‐to‐strong weighted Cohen's kappa values. All statistical measures were significant (*P* < 0.001), indicating that these variations were unlikely to be due to random chance. This is the first intra‐ and interobserver study within the literature assessing the performance of sonographers, sonologists and radiologists in the scoring of the IQ of gynecological TVS images.

The scoring system we created, which was designed to function like a task‐related checklist^28^, ^29^, was intended to assess IQ objectively^10^, ^13^, ^28^. However, our results suggest this system may not have been well‐enough defined, contributing to subjective interpretation among raters. The subjective nature of assessing ultrasound IQ by human operators is a well‐documented challenge^10^, ^19^, ^30^, ^31^. For instance, Papageorghiou *et al*.^31^ reported that experts agreed on the accuracy of fetal ultrasound images (as they adhered to established protocols) only 79.4% of the time. Although both Molloholli *et al*.^10^ and Wanyonyi *et al*.^30^ reported that utilizing an objective IQ scoring system resulted in better agreement between professionals than subjective assessment, other studies reported an element of subjectivity concerning IQ assessment, even with the utilization of a structured scoring system^13^. The inherent subjectivity in IQ assessment may explain our intraoperator results, which, although better than our interoperator agreement, were weaker than expected.

In the present study, we created a new IQ scoring system, as no published system existed within the literature that could be broadly applied suitably to TVS images. While three scoring systems were found within the literature relating to gynecological ultrasound^6^, ^11^, ^18^, these were either too specific to a single application^6^, ^11^ or assessed operator competence rather than IQ^18^. Our aim to make the scoring system applicable across various TVS scenarios may have affected its objectivity. While some similar systems in obstetric ultrasound have shown good results^10^, ^12^, ^18^, ^30^, ^32^, ^33^, ^34^, these are unlikely to translate into gynecological ultrasound, where normal anatomical appearances are more variable. Further refinement of the scoring instructions may improve levels of agreement in the future.

In addition to assessing IQ for quality assurance, IQ scoring systems can aid in teaching, training and providing feedback to professionals^11^, ^12^, ^32^, ^34^, with more experienced operators shown to produce higher scores using several published systems^4^, ^5^, ^11^, ^17^, ^18^. The utility of a scoring system as a teaching tool was highlighted in the study of Salomon *et al*.^11^, which found that scores improved significantly when trainee doctors performing gynecological ultrasound scans in the emergency department were given additional training. It remains unknown whether our system would be suitable for use as a training tool, which is an area for further research.

AI systems offer significant advantages over human assessment of IQ because they are not affected by human factors, such as fatigue^3^. The development of such systems, however, is not without difficulty. First, the subjectivity of IQ assessment often results in noisy image labeling, a problem AI tools typically struggle to manage^35^. Furthermore, AI tools need high volumes of labeled data, requiring precise image review by experts, such as the method undertaken in this study, which is both labor‐intensive and costly^12^, ^36^. To address these issues, innovative data‐labeling strategies need to be developed.

Most of the scoring systems we reviewed (Table 1) assessed the skill and competency of the operator as a means of ultrasound quality assurance, rather than the IQ of documented images in isolation^4^, ^7^, ^9^, ^13^, ^15^, ^16^, ^17^. In clinical practice, the skill and competence of sonographers are essential for ensuring examination accuracy and optimal patient outcomes. However, AI models assess ‘data quality’ (i.e. stored images and videos) independent of operator skill. As literature suggests that AI is already playing a significant role in the IQ assessment of other (non‐gynecological) ultrasound applications^19^, ^23^, it may be that we are perhaps entering a new paradigm in the assessment of ultrasound IQ, one that balances traditional human‐led methods alongside the emerging role of AI and digital systems.

This study has several strengths. A large dataset of TVS images acquired from various sonographers, clinics and ultrasound machines was used, resulting in statistically significant results. This also provided a variation in ‘data’, which must be considered when developing AI tools for either IQ assessment or image interpretation. Additionally, although all six raters are experienced professionals, by including sonographers, radiologists and sonologists, we were able to evaluate whether different professional perspectives affect how IQ is interpreted.

This work represents the first IQ assessment scoring system related broadly to gynecological imaging, applicable to both clinical practice and the design of AI tools. Despite its novelty, this study has several limitations, such as our decision to construct a scoring system from a review of published literature only, rather than a multicenter expert consensus or Delphi approach. Furthermore, our literature search was confined to studies on ultrasound only. Expanding our search to other imaging modalities reviewed more frequently offline may have identified concepts not explored previously in ultrasound. As all raters viewed images in unique locations on personal devices, environmental factors (e.g. screen settings and lighting) may have impacted image appearance. Although the system design underwent extensive review, we did not perform a pilot test of the system within the development phase, which could have allowed for further refinement and potentially improved the results of this study. Finally, although all raters were provided with uniform, written instructions, no calibration meeting was held prior to the study, which could have resulted in higher levels of agreement.

In conclusion, wide variation in inter‐ and intraobserver agreement was noted for IQ grading of gynecological TVS images utilizing our proposed scoring system. Although AI systems will likely be able to assess IQ more objectively than humans, the development of these systems will depend on human labeling, which will likely have labeling inconsistencies (creating noisy data) due to the inherently subjective nature of ultrasound IQ. As such, reliable methods of IQ scoring are essential for further progress in the development of AI systems in gynecological ultrasound. Additionally, consideration should be given to AI methods capable of accommodating noisy labeling.

## Supporting information


**Appendix S1** Various iterations of our objective scoring system throughout the development process

**Table S1** Median, mode and mean scores given by each rater at the first and second review for assessment of ultrasound image quality of all images (*n* = 150) and when grouped into uterine (Ut) (*n* = 50) and ovarian (Ov) images (*n* = 100)

## Data Availability

The data that support the findings of this study are available from the corresponding author upon reasonable request.
