# Supplementary material for: Intra‐ and interobserver agreement of proposed objective transvaginal ultrasound image‐quality scoring system for use in artificial intelligence algorithm development
Source: Ultrasound Obstet Gynecol. 2025 Jan 24;65(3):364–71. doi: 10.1002/uog.29178 (PMC11872342; doi:10.1002/uog.29178)
Supplement: Supplementary file 1 — Appendix S1 Various iterations of our objective scoring system throughout the development process [file UOG-65-364-s002.docx]

**Appendix S1**

Various iterations of our objective scoring system throughout development process.

*Iteration 1*

Below is a proposed quality assessment scoring tool which can be applied to any image acquired as part of a transvaginal ultrasound prior to being used in the development of an AI tool for diagnosis.

| **Score** | **1** | **2** | **3** | **4** | **5** |
| --- | --- | --- | --- | --- | --- |
| **Definition** | Image quality very poor | Image quality poor | Image quality suboptimal | Image quality adequate | Image quality optimal |

*Explanatory notes relating to each score*

1. A score of 1 represents **Image quality very poor**
   1. Anatomy cannot confidently be identified
   2. Incorrect anatomical structure assessed
   3. Sonographic optimisation too poor to interpret anatomical structures
   4. Image not reliable to interpret diagnosis
2. A score of 2 represents **Image quality poor**
   1. Anatomy difficult to confidently be identified
   2. Sonographic optimisation poorly performed making interpretation of anatomical structures difficult but not impossible
   3. Image not reliable to interpret diagnosis
3. A score of 3 represents **Image quality suboptimal**
   1. Anatomy recognisable
   2. Sonographic optimisation poorly performed
   3. Image interpretation for diagnosis possible
4. A score of 4 represents **Image quality adequate**
   1. Anatomy easily recognisable
   2. Sonographic optimisation well performed
   3. Limitations in image quality are minimal and have no bearing on ability of image to be interpreted for diagnosis
   4. Image interpretation for diagnosis easily possible
5. A score of 45represents **Image quality optimal**
   1. Anatomy easily recognisable
   2. Sonographic optimisation well performed with no limitations
   3. Image interpretation for diagnosis easily possible

*Iteration 2*

We are proposing a quality assessment scoring tool that can be applied to any image acquired as part of a transvaginal ultrasound. We also propose that this tool be implemented in the development of AI algorithm tools, with a minimum score of 3 for content input into an algorithm.

| **Score** | **1** | **2** | **3** | **4** | **5** |
| --- | --- | --- | --- | --- | --- |
| **Definition** | Image quality very poor | Image quality poor | Image quality suboptimal | Image quality adequate | Image quality optimal |

*Explanatory notes relating to each score*

1. A score of 1 represents **Image quality very poor**
   1. Anatomy cannot confidently be identified
   2. Incorrect anatomical structure assessed
   3. Sonographic optimisation too poor to interpret anatomical structures
   4. Image not reliable to interpret diagnosis
2. A score of 2 represents **Image quality poor**
   1. Anatomy difficult to confidently be identified
   2. Sonographic optimisation poorly performed making interpretation of anatomical structures difficult but not impossible
   3. Image not reliable to interpret diagnosis
3. A score of 3 represents **Image quality suboptimal**
   1. Anatomy recognisable
   2. Sonographic optimisation poorly performed
   3. Image interpretation for diagnosis possible
4. A score of 4 represents **Image quality adequate**
   1. Anatomy easily recognisable
   2. Sonographic optimisation well performed
   3. Limitations in image quality are minimal and have no bearing on ability of image to be interpreted for diagnosis
   4. Image interpretation for diagnosis easily possible
5. A score of 5represents **Image quality optimal**
   1. Anatomy easily recognisable
   2. Sonographic optimisation well performed with no limitations
   3. Image interpretation for diagnosis easily possible

*Iteration 3*

We are proposing a quality assessment scoring tool that can be applied to any image acquired as part of a transvaginal ultrasound. We also propose that this tool be implemented in the development of AI algorithm tools.

*The Scoring System*

| **0** | **1** | **2** | **3** | **4** | **5** |
| --- | --- | --- | --- | --- | --- |
| **Image Inaccurate** | **Image quality very poor** |  | **Image quality suboptimal** |  | **Image quality optimal** |
|  | *Correct anatomy not confidently recognisable* |  | *Correct anatomy recognisable* |  | *Correct anatomy easily recognisable* |
|  | *Some of the anatomical structure is not seen* |  | *Most of the anatomical structure is clearly seen* |  | *Entire anatomical structure clearly seen* |
|  | *Unacceptable image optimisation (depth, focus, gain)* |  | *Acceptable image optimisation (depth, focus, gain)* |  | *Good image optimisation (depth, focus, gain)* |
|  | *Unable to interpret image for diagnosis* |  | *Possible to interpret image for diagnosis* |  | *Easy to interpret image for diagnosis* |
|  | *Overall image clarity is poor* |  | *Overall image clarity is satisfactory* |  | *Overall image clarity is good* |

*Applying the scoring system*

- If an image is inaccurate for need (e.g. blank image, transabdominal image when transvaginal image required, etc), a score of 0 is applied
- All other images are assessed against the 5 factors in the table above relating to;
  - Correct depiction of anatomy
  - View of anatomical structure in field of view
  - Image optimisation (depth, focus and gain)
  - Ability of image to be interpreted for diagnosis of pathology
  - The overall clarity of the image
- A score of 1-5, which best represents the image, is allocated for each factor. Scores for all five factors are combined giving a total score of 5-25. This number is then divided by 5 to give an overall score to each image.

*Iteration 4 [Final system]*

We are proposing a quality assessment scoring tool that can be applied to any image acquired as part of a transvaginal ultrasound. We also propose that this tool be implemented in the development of AI algorithm tools.

Ultimately, a 3-point system was felt to be easier to interpret and be more objective. Additionally, the scoring options of 1-4 were used (rather than 0-3) as these translated best into our data storage platform.

A score of 1-4, which, in the opinion of the observer, best represents the image, is allocated for each factor. A selection of images representing various scores can be seen in figure 1.

Images are assessing for the five factors listed and assigned a score of 1-4 for each factor. A score of 4 represents optimal image quality while a score of 2 represent poor image quality.

| **Reject 1** | **2** | **3** | **4** |
| --- | --- | --- | --- |
| **Image Inaccurate** | **Image quality poor** | **Image quality suboptimal** | **Image quality optimal** |
|  | *Correct anatomy not confidently recognisable* | *Correct anatomy recognisable* | *Correct anatomy easily recognisable* |
|  | *Some of the anatomical structure is not seen* | *Most of the anatomical structure is clearly seen* | *Entire anatomical structure clearly seen* |
|  | *Unacceptable image optimisation (depth, focus, gain)* | *Acceptable image optimisation (depth, focus, gain)* | *Good image optimisation (depth, focus, gain)* |
|  | *Unable to interpret image for diagnosis* | *Possible to interpret image for diagnosis* | *Easy to interpret image for diagnosis* |
|  | *Overall image clarity is poor* | *Overall image clarity is satisfactory* | *Overall image clarity is good* |

Images are to be assigned a grade of 1 (quality poor), 2 (quality suboptimal) or 3 (quality optimal) based on the five criteria outline in table 1. If an image is inaccurate (e.g. the image should be demonstrating an ovary but is demonstrating the cervix, etc), this is a reject. The criteria by which the grades are assigned are;

1. Ability of anatomy to be recognised in image,
2. The clarity of the anatomical structure in field of view,
3. Image optimisation (depth, focus and gain),
4. Ability of image to be interpreted for diagnosis of pathology and
5. The overall clarity of the image.
