# Supplementary material for: Intra‐ and interobserver agreement of proposed objective transvaginal ultrasound image‐quality scoring system for use in artificial intelligence algorithm development
Source: Ultrasound Obstet Gynecol. 2025 Jan 24;65(3):364–71. doi: 10.1002/uog.29178 (PMC11872342; doi:10.1002/uog.29178)
Supplement: Supplementary file 2 — Table S1 Median, mode and mean scores given by each rater at the first and second review for assessment of ultrasound image quality of all images (n = 150) and when grouped into uterine (Ut) (n = 50) and ovarian (Ov) images (n = 100) [file UOG-65-364-s001.docx]

**Table S1** Median, mode and mean scores given by each rater at first and second review for assessment of ultrasound image quality of all images (*n*= 150) and when grouped by uterine (Ut) (*n*= 50) and ovarian (Ov) images (*n*= 100)

| Rater | First review | | | | | | | | | Second review | | | | | | | | |
| --- | --- | --- | --- | --- | --- | --- | --- | --- | --- | --- | --- | --- | --- | --- | --- | --- | --- | --- |
|  | Median | | | Mode | | | Mean | | | Median | | | Mode | | | Mean | | |
|  | All | Ut | Ov | All | Ut | Ov | All | Ut | Ov | All | Ut | Ov | All | Ut | Ov | All | Ut | Ov |
| M.L. | 4 | 4 | 4 | 4 | 4 | 4 | 3.5 | 3.6 | 3.4 | 4 | 4 | 4 | 4 | 4 | 4 | 3.4 | 3.6 | 3.3 |
| G.C. | 3 | 3 | 3 | 3 | 3 | 3 | 2.9 | 2.9 | 2.9 | 3 | 3 | 3 | 3 | 3 | 3 | 3.0 | 3.0 | 3.0 |
| S.M. | 2 | 2 | 2.5 | 2 | 2 | 2 | 2.6 | 2.5 | 2.6 | 3 | 3 | 3 | 3 | 3 | 3 | 2.8 | 2.7 | 2.9 |
| C.P. | 4 | 4 | 3.5 | 4 | 4 | 4 | 3.4 | 3.6 | 3.3 | 4 | 4 | 4 | 4 | 4 | 4 | 3.5 | 3.7 | 3.4 |
| G.L. | 3.5 | 4 | 3 | 4 | 4 | 4 | 3.2 | 3.4 | 3.1 | 3 | 4 | 3 | 4 | 4 | 4 | 3.2 | 3.4 | 3.0 |
| S.K. | 3 | 3 | 3 | 4 | 4 | 4 | 3.1 | 3.2 | 3.1 | 3 | 4 | 3 | 4 | 4 | 4 | 3.0 | 3.2 | 3.0 |
